# A new local flap technique for closing long-axis skin defects: the cyclist flap procedure

**DOI:** 10.1308/rcsann.2023.0077

**Published:** 2023-12-01

**Authors:** Ö Öcük

**Affiliations:** Kahramanmaraş Necip Fazıl City Hospital, Turkey

**Keywords:** Cyclist flap, Local flap, Long-axis, Skin defect, New procedure

## Abstract

**Background:**

Local flaps are commonly used during the treatment of skin tissue defects. Although there are many available procedures for the closure of triangular, circular and rhomboid-shaped defects, long-axis elliptical skin defects lack treatment options. To address this problem, a new local flap design called the cyclist flap procedure (CFP) was developed, so called because it resembles the silhouette of a person riding a bicycle.

**Methods:**

The CFP was performed in 29 patients aged 9–81 years in 2021–2022. The defects were localised in the sacral region (1), upper extremities (3), lower extremities (3), and head and neck regions (22). Closures of skin defects due to pressure ulcers (one patient) and after tumour excision (28 patients) were performed, and the patients were followed up for 12–20 months.

**Results:**

Only minor complications were observed in two patients. During follow-ups, no secondary surgery was required due to dog-ear, trapdoor, recurrence or revision. Furthermore, the aesthetic results related to the skin lines and scar size were acceptable.

**Conclusion:**

The CFP is a new, easy-to-apply and advantageous procedure for the closure of long-axis elliptical and oval skin tissue defects.

## Introduction

Local flaps, which are primarily used for the closure of skin tissue defects, are one of the most frequently used techniques in plastic surgery. Many local flap techniques have been described.^1,2^ New flap designs are occasionally created based on the size, shape and localisation of the defect in the body. An ideal local flap is associated with features such as a similar tissue appearance, no tension on the skin after closure of the defect, no dog-ear deformity, no requirement of intact tissue excision, no disruption of the anatomical lines of the region, absence of excessive scarring and distribution of closure tension to all areas around the defect.^1,3–5^ Many local flap designs have been described for the closure of round, triangular and rhomboid-shaped defects; fewer options are available for elliptical or oval tissue defects with a long axis. Here, a new local flap procedure, named the cyclist flap procedure (CFP), is described for the closure of long-axis skin tissue defects using a combination of z-plasty and rotation flap. Its name derives from its resemblance to the silhouette of a person riding a bicycle. The use of analogy to describe local flap shape is both catchy and practical. Such imagery has been used previously to describe local flap shape, such as the jumping man, reading man or omega flap.^2–5^

## Methods

The procedure was performed in 29 patients in the Plastic Reconstruction and Aesthetic Surgery Clinic at Kahramanmaraş Necip Fazil City Hospital in 2021–2022. Consent was obtained from the local ethics committee (consent number 2021/16-01). The age of the patients (19 males and 10 females) included in this study ranged from 9 to 81 years. They had defects in the sacral region (1), lower (3) and upper (3) extremities, and head and neck regions (22). The CFP was used for the closure of defects due to decubitus ulcers (1 patient) or after tumour excision (28 patients). The smallest and largest defect sizes observed were 1.5×0.9 and 9×6cm in the nasal ala and thigh region, respectively. After the procedure, the patients were followed up for 12–20 months (Table 1).

**Table 1:** Patients undergoing cyclist flap procedure (CFP) and clinical follow-up parameters

Patients	Age (years)	Gender	Locality	Diagnosis	Defect Size (cm)	Complication	Follow-up (month)
1	67	M	Arm	Skin CA	5×4	No	14
2	77	M	Scalp	Skin CA	3×2	No	14
3	19	M	Scalp	Nevus sebaceous	8×4	No	15
4	77	M	Hand dorsum	Keratoacanthoma	5×3	No	14
5	67	M	Nasal dorsum	Skin CA	2.5×1.4	No	13
6	70	F	Zygomatic	Skin CA	3×2	No	14
7	68	M	Preauricular	Skin CA	5×3	No	15
8	39	F	Nasal ala	Skin CA	2.6×1.9	No	13
9	80	F	Nasal dorsum	Skin CA	2.8×2	No	16
10	69	F	Nasal ala	Skin CA	1.5×0.9	Partial tip necrosis	16
11	84	F	Thigh	Skin CA	9×6	No	14
12	75	F	Nasal dorsum	Skin CA	2×1.2	No	15
13	9	F	Sacral	Decubitus ulcer	4×2	No	12
14	61	M	Nasal tip	Skin CA	1.5×1.1	No	12
15	40	M	Hand finger	Pyogenic granuloma	2×1.3	No	16
16	78	M	Fronto zygomatic	Skin CA	4×3	No	15
17	68	M	Scalp	Skin CA	3.5×2.6	No	14
18	81	M	Foot	Dysplastic nevus	3×2	Wound dehiscence	12
19	27	F	Hand finger web	İntradermal nevus	1.7×1.2	No	15
20	77	M	Zygomatic	Skin CA	4×3	No	12
21	74	M	Nasal dorsum	Skin CA	3.8×2.1	No	15
22	55	M	Nasal dorsum	Skin CA	1.6×1.2	No	14
23	64	M	Preauricular	Seborrheic keratosis	4×3	No	15
24	68	M	Infraorbital	Skin CA	4×2	No	15
25	71	M	Scalp	Skin CA	4×3	No	14
26	36	M	Pretibial	Pyogenic granuloma	3×1.8	No	20
27	55	F	Nasal tip	Skin CA	1.5×1.1	No	15
28	71	F	Nasal ala	Skin CA	1.6×1.1	No	16
29	35	M	Scalp	Skin CA	3.2×2.1	No	20

### Surgical technique

**Figure 1:**
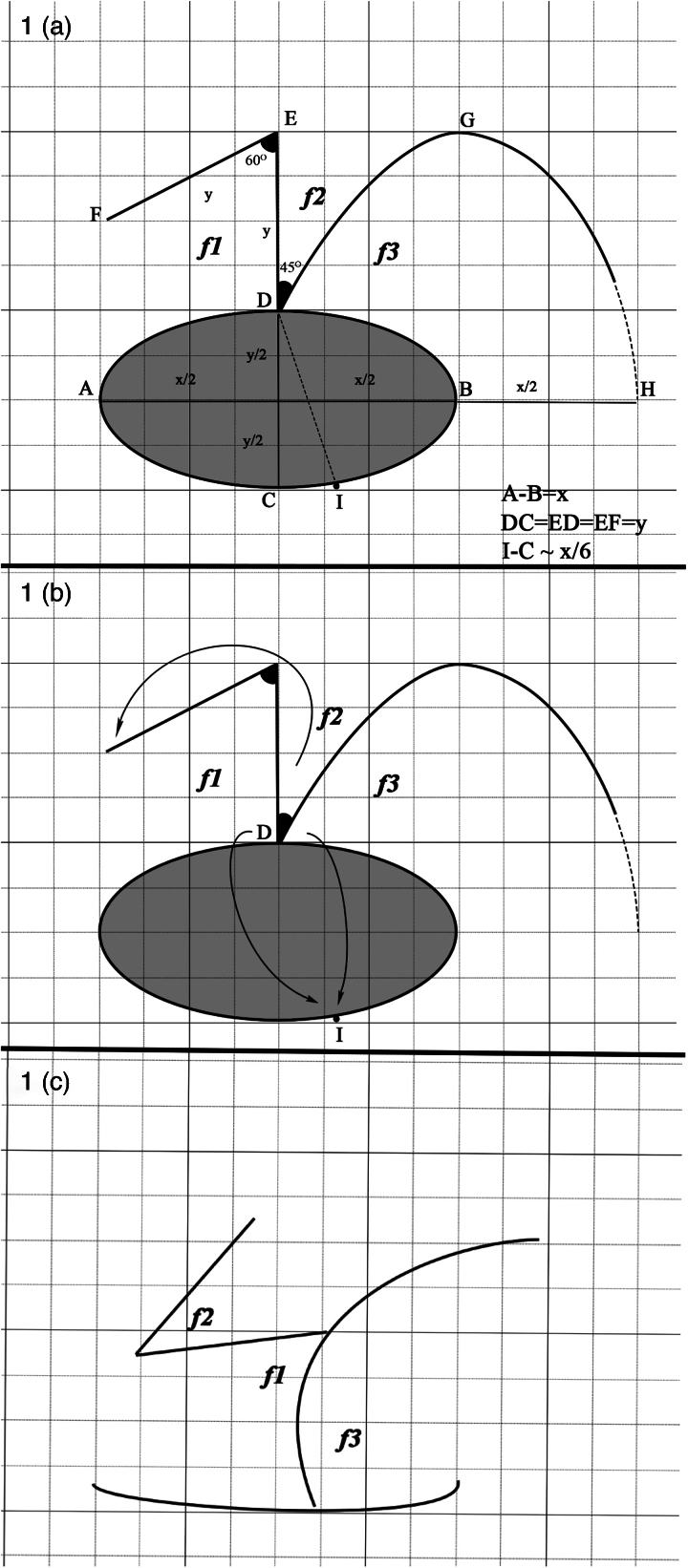
Drawing of flap plan. (a) drawing plan of the cyclist flap procedure (CFP) to close the elliptical defect (*f1, f2, f3* flaps); (b) drawing showing the transposition and rotation direction of the flaps; (c) scar lines that will occur when the flaps are adapted to their new places.

**Figure 2:**
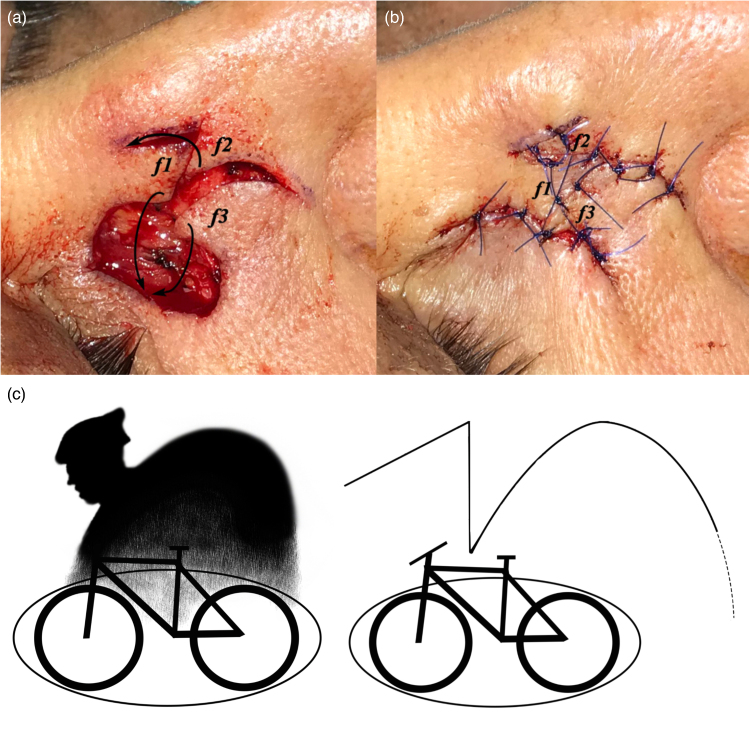
Display of the cyclist flap procedure (CFP) on the patient. (a) Appearance after excision and flap incisions are made. The *f1, f2, f3* flaps are adapted to their new positions by moving them in the directions of the arrows. (b) The appearance of the incision and scar line after the adaptation of the flaps. (c) Illustration showing the tissue defect looking like a bicycle, and the new procedure represented by an image of a person riding a bicycle.

The CFP consists of three flaps using a combination of z-plasty and a rotation flap. It can be easily used for the closure of elliptical skin defects with long and short axes. As seen in Figure 1a, the distance to the short axis length (CD=y) perpendicular to the long axis (AB=x) of the defect is marked (ED). A second marking of the same length (EF) is made at an angle of 60°. The G point, which is the intersection point of the projections made parallel to the long and short axes from points B and E, is determined. The distance (x/2), which is half the length of the long axis, is determined as the H point by measuring as the continuation of the long axis. The DGH line, which forms a 45° angle with the ED line, proceeds to form a parabolic curve and forms the rotation flap boundary (Figure 1a). After drawing, incisions are made along the ED and EF lines. The incision is made starting from point D on the DGH curve, but is not advanced all the way to the H point; it is terminated at approximately y/2 distance before reaching point H (the distance marked as a dotted line near point H of the DGH curve). The extension of this incision to the H point is decided according to the skin tension and structure in the working area. It may not always be necessary to extend the incision to the H point. The *f1*, *f2* and *f3* fasciocutaneous flaps are harvested. The D point, which is the intersection point of the *f1* and *f3* flaps, is adapted to point I on the opposite side of the defect with a suture (Figure 1b). Point I is approximately a distance of x/6 from point C. Although not a definite rule, it has been shown that the flap forms the most comfortable tension line at this point (point I) in cases adapted to this point. After the adaptation at this point is achieved, the *f2* flap is advanced to close the donor area of the *f1* flap and its adaptation is achieved with sutures. Finally, the *f1* flap is adapted to the edge of the *f3* flap to the point where appropriate tension is achieved (Figures 1c and 2a,b). Figure 2c illustrates how this flap resembles a cyclist.

### Case 1

**Figure 3:**
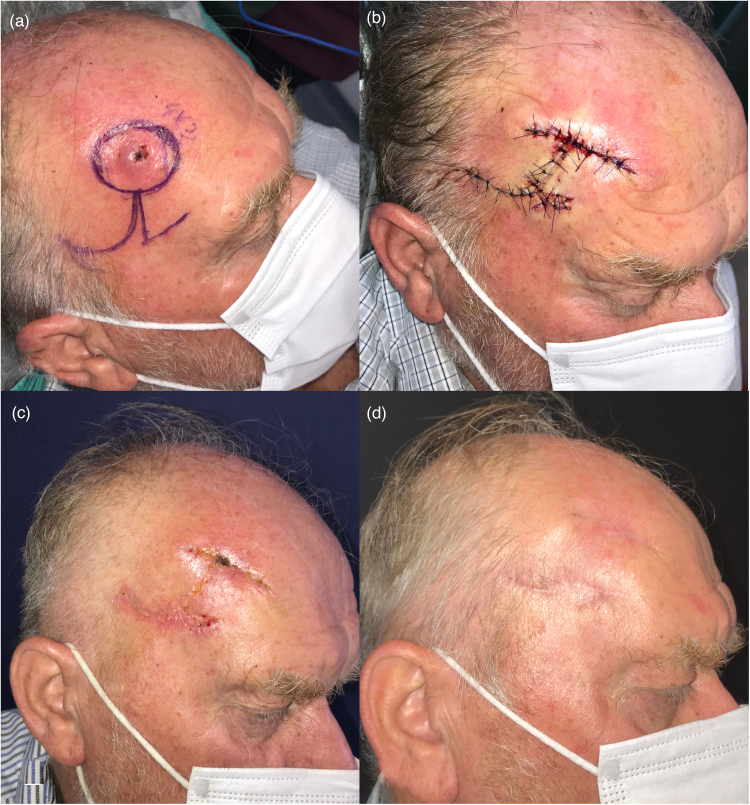
Case 1. (a) Preoperative (preop) view and drawing plan. (b) View of the postoperative (postop) incision line. (c) Early postop period (second week). (d) Late postop period (third month).

A 71-year-old man was admitted to our clinic with an ulcerated skin tumour in the frontoparietal region. After drawing at the safe margin, it was deemed appropriate to close the defect with CFP. The defect size was 4×3cm. The procedure was performed without complications by elevating the flaps subperiosteally. Furthermore, no complications occurred during the postoperative follow-up of the patient. The pathological report showed a well-differentiated squamous cell carcinoma, and recurrence or any other long-term complications were not observed. The final scars were acceptable and at the appropriate aesthetic level (Figure 3).

### Case 2

**Figure 4:**
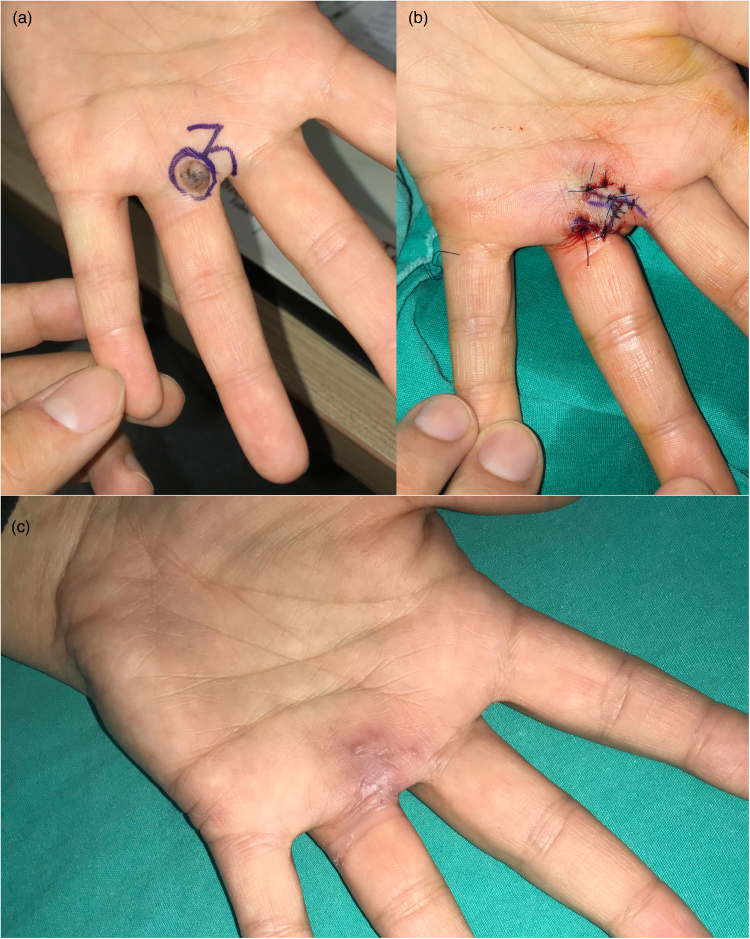
Case 2. (a) Preoperative (preop) view and drawing plan. (b) View of the postoperative (postop) incision line. (c) Late postop period (second month).

A 27-year-old female patient presented with a pigmented skin tumour at the metacarpopharyngeal joint level of the left-hand fourth finger volar. Considering that the joint area was involved and that primary closure of the defect might cause contraction, a CFP was considered appropriate. The size of the defect was 1.6×1.1cm. During the postoperative follow-up, contracture was not observed, which avoided restricted joint movement and a narrow web space. Pathological examination identified an intradermal naevus. No recurrence or scar formation was observed during long-term follow-ups (Figure 4).

### Case 3

**Figure 5:**
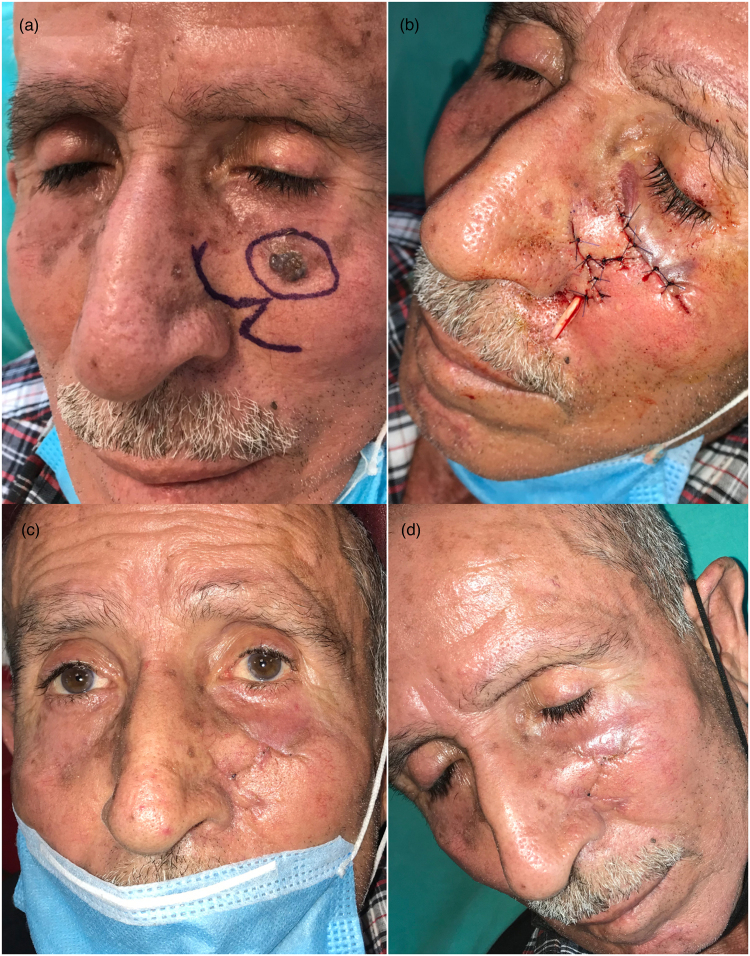
Case 3. (a) Preoperative (preop) view and drawing plan. (b) View of the postoperative (postop) incision line. (c) Early postop period (second week). (d) Late postop period (second month).

A 68-year-old man was admitted to our clinic with an ulcerated skin tumour in the left infraorbital region. A drawing was prepared with a safe margin; the defect area was 4×2cm. After excision of the tumour, a CFP was performed. A risk of developing ectropion was identified owing to the proximity of the defect area to the lower eyelid; however, the CFP evenly distributed the skin tension area and neutralised the risk. Pathological examination identified basosquamous cell carcinoma. No recurrence or complications were encountered at follow-up (Figure 5).

## Results

All CFP were performed under local anaesthesia by a single surgeon. One patient showed partial necrosis of the *f3* flap, while another developed partial dehiscence at the suture line; the local necrotic area was closed with a secondary debridement, but no procedure was performed in the patient with suture dehiscence. Dog-ear or trapdoor was not observed in any of the patients. Intact skin excision was not performed in any patient. None of the patients required secondary surgery or revision. Furthermore, no recurrence events were observed during the follow-up period in patients requiring tumour excision, with no requirements for re-excision. During follow-up, none of the patients showed tension or scar lines on the skin that would have disrupted the anatomical lines.

## Discussion

Z-plasty and rotation flaps have been routinely used in local flap procedures in plastic surgery.^1,5^ Various local flap designs have been developed using many combinations of unique skin defects.^1–9^ Skin defects in various parts of the body are closed quickly and practically with these newly designed flaps owing to their unique advantages. Thus, the available methods are diverse and offer a wide scope for reconstruction.

For the closure of round, triangular and rhomboid-shaped defects, many procedures exist,^2–4,6–9^ but fewer options are available for elliptical lesions with long axes, which require an intact skin excision to reshape defects into a round, triangular or rhomboid shape, depending on the location and shape of the original defect. However, it is undesirable to excise intact skin to close the defect.^2^ The CFP, which is designed to avoid this situation, does not require healthy skin excision, and also offers many advantages over other options available to treat elliptical defects.

The Dufourmentel flap, which is a rhomboid flap modification,^1,8^ is the most basic flap option for the closure of elliptical defects with a long axis; however, this still requires a rhomboid design and, furthermore, it requires a blood supply from a single site. The CFP eliminates this limitation; blood flow is safer as three separate flaps receive blood flow from three separate areas. In the Dufourmentel flap procedure, a single tension line is formed on the skin while closing the donor area. It is preferable and advantageous to distribute the tension homogeneously in local flaps.^4,5^ In CFP, the tension from the skin is distributed more evenly to the defect area owing to the mobility of the three flaps. Cuono^6^ and Becker^7^ flaps are also used to close long-axis defects but also require creation of rhomboid shapes similar to the Dufourmentel flap. These two flaps utilise two opposing z-plasties. Although these two flaps distribute the skin tension in both areas, they are difficult to use in marginal areas such as the nasal rim and infraorbital region, as flap dissection is required on both sides of the defect. Unilateral planning in the CFP is more suitable for closing tissue defects in these areas.

Advancement flaps are also used to close elliptical defects.^1^ In the most commonly used V-Y advancement flap, the flap is dependent on a certain perforator coming from the base. Since the mobility of the advancement flaps is limited, the flap should be used in areas of the body where tissue elasticity is high. Hence, these flaps have limited applications in areas such as the head and neck. Bilateral H advancement flaps are planned randomly, but still depend on skin elasticity. These flaps also require the removal of intact skin with Burow’s triangle excision because of dog-ear formation in the donor area. Further, these flaps create very large incision areas and lead to a large number of scar lines. The keystone flap described by Behan^10^ is also associated with the following disadvantages: 1) creation of large incision areas; 2) the long axis of the defect should be parallel to the direction of movement of the skin; and 3) the limitation of use in the head and neck region. Another method that can be used to close elliptical defects is the oval omega advancement flap described by Arpaci *et al*,^11^ but being an advancement flap, the method is associated with similar disadvantages. Another disadvantage of this method is the involvement of de-epithelisation of the intact triangular tissue where a much larger skin island, as compared with that of the defect, is required for flap design. In contrast, during the CFP, it is not necessary to de-epithelise the intact tissue. In addition, skin elasticity is not the primary priority and there is no perforator dependency. Bipedicled flaps can be used for closure of elliptical defects, but they require skin grafts for the closure of the donor site, hence creating a significant disadvantage owing to additional donor site defects and an aesthetically undesirable appearance. Use of bipedicled advancement flaps is specific to elliptical defects in the nasal supratip region.^12^ In this flap, it may be necessary to remove intact skin tissue, or there is a very large dissection area extending to the glabella. The L-shaped flap, which is an old method for closure of long-axis triangular defects, also presents an option for the closure of elliptical defects.^13^ The associated disadvantages include the necessity of healthy tissue excision to make the defect triangular and blood circulatory safety being critical.

Rotation flaps can also be used for the closure of elliptical defects. In this procedure, it is necessary to change the shape of the defect; however, the removal of intact skin islands such as the Burow’s triangle is undesirable. Further, the rotation arc is generally four times the diameter of the defect, limiting its application.^1^ Such wide arcs of rotation cause extensive dissection and a large scar line, while skin grafts for donor site closure are required with short rotation arcs. The CFP eliminates these disadvantages with modifications such as short rotational arcs (hence less scarring and dissection) and no skin graft requirements for donor site closure.

In some ways, the CFP flap is similar to the reading man flap.^2^ However, the reading man flap is mostly used for closure of circular defects and does not have an additional rotation flap. The advantage of the rotation flap here is to provide a more professional closure of the long axis defect. In addition, the planning and placement of flaps in CFP differs from that of the reading man flap. This situation paves the way for many combinations and innovations by using basic plastic surgery techniques more creatively.

The incidence of scar traces is low in local flaps planned in accordance with the skin lines. In the CFP, planning in accordance with the skin contours of the nose rim, nasolabial and malar regions yielded clinically better aesthetic results. A trapdoor deformity is a contraction of the scar line during healing encountered in V- or U-shaped incisions.^13^ During CFP, trapdoor deformity was not observed in any of the patients. Further, dog-ear formation that may occur during flap planning creates an undesirable appearance on the flap line during the healing process; however, we did not encounter dog-ear development in the tissues repaired using the CFP.

## Conclusion

CFP is a new alternative for elliptical or oval-shaped skin tissue defects with a long axis. The procedure allowed defect closure without excision of intact tissues for shape modifications. This procedure, which can be visualised as an image of a person riding a bicycle, can potentially act as an aid to the memory when planning in clinical situations. Furthermore, in this procedure, the planning of the flap is very simple and suitable for many parts of the body. Finally, the procedure can serve as a guide for other studies.
